# Curcumin Nanoparticles Ameliorate ICAM-1 Expression in TNF-α-Treated Lung Epithelial Cells through p47 *^phox^* and MAPKs/AP-1 Pathways

**DOI:** 10.1371/journal.pone.0063845

**Published:** 2013-05-09

**Authors:** Feng-Lin Yen, Ming-Horng Tsai, Chuen-Mao Yang, Chan-Jung Liang, Chun-Ching Lin, Yao-Chang Chiang, Hui-Chun Lee, Horng-Huey Ko, Chiang-Wen Lee

**Affiliations:** 1 Department of Fragrance and Cosmetic Science, College of Pharmacy, Kaohsiung Medical University, Kaohsiung, Taiwan; 2 Division of Neonatology and Pediatric Hematology/Oncology, Department of Pediatrics, Chang Gung Memorial Hospital, Yunlin, Taiwan; 3 Department of Physiology and Pharmacology, Chang Gung University, Kwei-San, Tao-Yuan, Taiwan; 4 Department of Anatomy and Cell Biology, College of Medicine, National Taiwan University, Taipei, Taiwan; 5 Graduate Institute of Natural Products, College of Pharmacy, Kaohsiung Medical University, Kaohsiung, Taiwan; 6 School of Pharmacy, College of Pharmacy, Kaohsiung Medical University, Kaohsiung, Taiwan; 7 Center for Drug Abuse and Addiction, China Medical University Hospital, Taichung, Taiwan; 8 China Medical University, Taichung, Taiwan; 9 Division of Basic Medical Sciences, Department of Nursing, and Chronic Diseases and Health Promotion Research Center, Chang Gung Institute of Technology, Chia-Yi, Taiwan; University of Illinois College of Medicine, United States of America

## Abstract

Upregulation of intercellular adhesion molecule-1 (ICAM-1) involves adhesions between both circulating and resident leukocytes and the human lung epithelial cells during lung inflammatory reactions. We have previously demonstrated that curcumin-loaded polyvinylpyrrolidone nanoparticles (CURN) improve the anti-inflammatory and anti-oxidative properties of curcumin in hepatocytes. In this study, we focused on the effects of CURN on the expression of ICAM-1 in TNF-α-treated lung epithelial cells and compared these to the effects of curcumin water preparation (CURH). TNF-αinduced ICAM-1 expression, ROS production, and cell-cell adhesion were significantly attenuated by the pretreatment with antioxidants (DPI, APO, or NAC) and CURN, but not by CURH, as revealed by western blot analysis, RT-PCR, promoter assay, and ROS detection and adhesion assay. In addition, treatment of TNF-α-treated cells with CURN and antioxidants also resulted in an inhibition of activation of p47 *^phox^* and phosphorylation of MAPKs, as compared to that using CURH. Our findings also suggest that phosphorylation of MAPKs may eventually lead to the activation of transcription factors. We also observed that the effects of TNF-α treatment for 30 min, which includes a significant increase in the binding activity of AP-1 and phosphorylation of c-jun and c-fos genes, were reduced by CURN treatment. *In vivo* studies have revealed that CURN improved the anti-inflammation activities of CURH in the lung epithelial cells of TNF-α-treated mice. Our results indicate that curcumin-loaded polyvinylpyrrolidone nanoparticles may potentially serve as an anti-inflammatory drug for the treatment of respiratory diseases.

## Introduction

Lung inflammation is a critical event in the pathogenesis of various diseases, including asthma, chronic obstructive pulmonary disease (COPD), severe acute respiratory syndrome (SARS), and cancer [1], [2]. Increased levels of adhesion molecules might contribute to the recruitment of PMNs to the various regions of the lung during the inflammation process [3]. Intercellular adhesion molecule-1 (ICAM-1) is one of the most important adhesion molecules; it mediates the tight adhesiveness of PMNs, facilitates PMN migration across the vascular endothelium barrier, and interacts with lung epithelium [4]. Reduced expression of ICAM-1 in the lung epithelium is currently considered as a novel therapeutic approach in the management of respiratory diseases. Previously, upregulation of ICAM-1 by cytokines has been shown to be regulated by the phosphorylation of three MAPKs, p38, JNK1/2, and Erk1/2, as well as transcription factors such as nuclear factor κB (NF-κB) and activator protein 1 (AP-1) in lung epithelial cells [5], [6]. Recently, increasing evidence have shown that NADPH oxidase (NOX)-derived ROS generation can modify signaling through the oxidation of reactive cysteines within certain cell signaling molecules, especially MAPKs or transcription factors such as NF-κB and AP-1 [7], [8]. The NOX family of ROS-producing enzymes has been increasingly recognized as a major source of ROS in cells. At least seven NOX isoforms have been identified, namely, NOX1 to 5 and Duox1 and Duox2; each isoform is distinguished according to tissue distribution, structure, and mode of activation [9], [10]. NOX2, now known as gp91*^phox^*, is the first isoform discovered and is the most extensively studied NOX member. Activation of NOX2 is partially regulated by the assembly of the catalytic gp91*^phox^* subunit with the regulatory subunits (p22*^phox^*, p47*^phox^*, p40*^phox^*, and small GTPase, Rac). The enzyme catalyzes the transfer of electrons from NADPH to molecular oxygen, generating superoxide anions and other forms of ROS as by-products. While NOX2 is most highly expressed in phagocytes, expression has also been detected in various cells, including lung epithelium [11], [12]. Moreover, elevated levels of ROS have been shown to activate signal transduction cascades and to drive the expression of adhesion molecules such as ICAM-1 and VCAM-1 in endothelial cells, epithelial cells, fibroblasts, and astrocytes [13], [14]. Thus, blockade of ROS production may be selectively targeted to reduce neutrophil adhesion through adhesion molecule suppression and attenuation of the inflammatory responses in lung diseases.

Curcumin, a naturally occurring phenolic compound found in *Curcuma longa*, has been shown to possess potent anti-oxidant, anti-inflammatory, and anti-tumor activities. It can regulate multiple transcription factors, cytokines, growth factors, kinases, and other enzymes [15], [16]. However, curcumin has low bioavailability and this is attributable to its poor aqueous solubility and dissolution properties. Nanonization serves as a mechanism to circumvent this limitation, facilitating in the production of curcumin nanoparticles that are highly water-soluble. Increasing water solubility may also increase the biodistribution and bioavailability of curcumin, and potentially slow down its rapid metabolism and systemic elimination [17], [18]. We have utilized polyvinylpyrrolidone (PVP) for the development of a novel curcumin nanoparticle system (CURN). PVP is a strong hydrophilic polymer that has been extensively utilized in improving the solubility and bioavailability of compounds with poor water-solubility; it delays the crystallization of compounds by forming molecular adducts [19]. Our previous study demonstrated that the physicochemical properties of curcumin using the PVP nanoparticle system have improved; these include a reduction in the compound particle size, the formation of a high-energy amorphous state, and the induction of intermolecular hydrogen bonding, which altogether enhanced its water solubility and drug release. Moreover, the improvement in drug release has enhanced the antioxidant and anticancer effects of CURN in hepatocytes compared to the CUR water preparation.

This study evaluated the effects of CURN on TNF-α-induced ICAM-1 expression in lung epithelial cells and subsequent lung inflammation in mice, in comparison with the inhibitory effect of curcumin in water (CURH). These effects are associated with the inhibition of NOX2-derived ROS generation, decreasing MAPKs activity, and AP-1 transcription factor binding activity.

## Materials and Methods

### Materials

Recombinant human TNF-α was from R&D System (Minneapolis, MN, USA). Anti-ICAM-1, anti-p47*^phox^*, anti-phospho c-fos and anti- phospho c-jun antibodies were from Santa Cruz (Santa Cruz, CA, USA). Anti-phospho p47*^phox^* antibody was from Assay Biotechnology Company (Sunnyvale, CA, USA). Anti-phospho p42/p44 MAPK, anti-phospho p38 MAPK, and anti-phospho JNK1/2 antibodies were from Cell Signaling (Danver, MA, USA). diphenyleneiodonium chloride (DPI), U0126, SB202190, SP600125, Tanshinone IIA, curcumin were from Biomol (Plymouth Meeting, PA, USA). Bicinchoninic acid (BCA) protein assay kit was from Pierce (Rockford, IL, USA). Curcumin-loaded polyvinylpyrrolidone nanoparticles (CURN) was were prepared as described previously [19]. CM-H_2_DCFDA was from Molecular Probes (Eugene, OR, USA). Apocynin (APO) was from ChromaDex (Santa Ana, CA, USA). Luciferase assay kit was from Promega (Madison, WI, USA). N-acetyl-L-cysteine (NAC) and other chemicals were from Sigma (St. Louis, MO, USA).

### Cell culture

A549 cells, a human lung epithelial cell carcinoma, were purchased from Food Industry Research and Development Institute (Taiwan) and cultured in DMEM/F-12 supplemented with 10% FBS and antibiotics (100 U/ml penicillin G, 100 mg/ml streptomycin, and 250 ng/ml fungizone) at 37°C in a humidified 5% CO_2_ atmosphere. When the cultures reach confluence (5 days), cells were treated with 0.05% (w/v) trypsin/1mMEDTA for 5 min at 37°C. The cell suspension diluted with DMEM/F-12 containing 10% FBS to a concentration of 10^5^ cells/ml. The cell suspension was plated onto (1 ml/well) 12-well culture plates and (10 ml/dish) 10-cm culture dishes for the measurement of protein expression and mRNA accumulation, respectively. Culture medium was changed after 24 h and then every 3 days.

### Western blot analysis

Growth-arrested A549 cells were incubated with TNF-α at 37°C for the indicated times. The cells were washed, scraped, collected, and centrifuged at 45000×*g* at 4°C for 1 h to yield the whole cell extract, as previously described [20]. Samples were denatured, subjected to SDS-PAGE using a 12% running gel, and transferred to nitrocellulose membrane. Membranes were incubated with anti-ICAM-1, anti-c-fos, anti-c-jun, anti-phospho p42/p44 MAPK, anti-phospho p38 MAPK, and anti-phospho JNK1/2 antibodies antibody for 24 h, and then membranes were incubated with an anti-mouse or rabbit horseradish peroxidase antibody for 1 h. The immunoreactive bands detected by ECL reagents were developed by Hyperfilm-ECL.

### RT-PCR analysis

Total RNA was isolated with Trizol according to the protocol of the manufacturer. The cDNA obtained from 0.5 µg total RNA was used as a template for PCR amplification as previously described (Lee et al., 2008). The primers used were as follows: 5′-TGACGGGGTCACCCACACTGTGCCCATCTA-3′ (sense) and 5′-CTAGAAGCATTTGCGGTGGACGATG-3′ (anti-sense) for β-actin; 5′-CAAGGGGAGGTCACCCGCGAGGTG-3′ (sense) and 5′-TGCAGTGCCCATTATGACTG-3′ (anti-sense) for ICAM-1.

### Measurement of ICAM-1 luciferase activity

The human ICAM-1 (pIC-339/0)/firefly luciferase was kindly provided by Dr. P. T. van der Saag (Hubrecht Laboratory, Utrecht, The Netherlands). ICAM-1-luc activity was determined as previously described [4] using a luciferase assay system (Promega, Madison, WI, USA). Firefly luciferase activities were standardized for β-gal activity.

### Isolation of cell fraction

A549 cells were harvested, sonicated for 10 s at output 1.5 with a sonicator (Misonix, Farmingdale, NY), and centrifuged at 8000 rpm for 15 min at 4°C. The pellet was collected as the nuclear fraction, which used for c-fos and c-jun assay. The supernatant was centrifuged at 14000 rpm for 60 min at 4°C to yield the pellet (membrane fraction) and the supernatant (cytosolic fraction), which used for p47 *^phox^* assay.

### Isolation of bronchoalveolar lavage (BAL) fluid

BAL fluid was determined as previously described [21]. Mice were injected with TNF-α at a dose of 0.75 mg/kg and sacrificed 24 h later. BAL fluid was administered through a tracheal cannula using 1-ml aliquots of ice-cold phosphate-buffered saline (PBS) medium. BAL fluid was centrifuged at 500 g at 4°C, and cell pellets were washed and resuspended in PBS. Leukocyte count was determined by a hemocytometer.

### Transient transfection with siRNAs

SMARTpool RNA duplexes corresponding to human p47*^phox^*(SC-29422), NOX2 (gp91*^phox^*, SC35503) and scrambled siRNA(SC-37007) were from Santa Cruz Biotechnology(CA, USA).Transient transfection of siRNAs was carried out using transfection reagent. siRNA (100 nM) was formulated with transfection reagent according to the manufacturer's instruction. The transfection efficiency (approximately 60%) was determined by transfection with EGFP.

### Measurement of intracellular ROS accumulation

Detection of ROS (O2 ^•−^ and H2O2) production. The effect of CURN on superoxide anion (O2 ^•^^−^) and H2O2 production by A549 cells were determined by a fluorimetric assay using dihydroethidium (DHE) and Amplex red as the probe, respectively [8]. Confluent A549 cells were incubated with or without 50 µM CURN and CURH for 1 h or 10 µM DPI for 1 h. A549 cells were incubated with 20 µM DHE for 20 min or with 50 µM Amplex red/HRP for 10 min at 37°C, and then 30 ng/ml TNF-α was added to the well for the indicated time. The fluorescence density (relative fluorescence units) was detected at 588 nm/630 nm and 544 nm/590 nm for excitation/emission, respectively, for ethidium corresponding to O2 ^•−^ and resorufin to H2O2, using a multidetection reader (SpectraMax M5; Molecular Devices, Sunnyvale, CA, USA).

### Determination of NADPH oxidase activity by chemiluminescence assay

Plasma membrane preparation, NOX activity assay, and Western blot analysis of p47 *^phox^* and NOX2 (p91 *^phox^*). The cytosolic and plasma membrane fractions were prepared as described previously with modification [8]. Briefly, A549 cells were lysed in lysis buffer A (20 mM Tris–HCl, 10 mM EGTA, 2 mM EDTA, 2 mM dithiothreitol, 1 mM phenylmethylsulfonyl fluoride (PMSF), 25 µg/ml aprotinin, and 10 µg/ml leupeptin). Cell lysates were centrifuged at 16,000 g for 20 min at 4°C. The supernatant was collected and designated the cytosolic fraction. The pellets were resuspended in lysis buffer B (0.5% sodium dodecyl sulfate, 1% NP-40, 1 mM Na3VO4, 1mMNaF, 1 mMPMSF, 25 µg/ml aprotinin, and 10 µg/ml leupeptin).Western blot analysis for p47 *^phox^* and NOX2 (p91 *^phox^*) were performed on the plasma membrane fractions as described above, using a monoclonal mouse antibody against 47 *^phox^* and NOX2 (p91 *^phox^*). For NOX activity assay, A549 cells were lysed in lysis buffer containing 20 mM monobasic potassium phosphate (pH 7.0), 1 mM EGTA, 10 µg/ml aprotinin, 0.5 µg/ml leupeptin, 0.5 mM PMSF. Plasma membrane fractionswere measured in a lucigenin chemiluminescence assay using 1 mM lucigenin (Sigma) and 5 mM NADPH (Sigma) as described.

### Electrophoretic mobility-shift assay (EMSA)

The preparation of nuclear protein extracts and the EMSA conditions have been described previously [8]. Nuclear proteins were extracted using NE-PER reagent (Pierce, Rockford, IL, USA) according to the manufacturer's protocol. The AP-1 binding activity of equal amounts (10 µg) of nuclear protein was analyzed using a LightShift chemiluminescence EMSA kit (Pierce). The synthetic double-stranded oligonucleotides used as the probes in the gel-shift assay were 5′-CGCTTGATGAGT-CAGCCGGAA-3′ and 3′-GCGAACTACTCAGTCGG- CCTT-5′ for AP-1.

### Adhesion assay

A549 cells were grown to confluence in 6-well plates, incubated with TNF-α for 24 h, and then adhesion assays were performed. Briefly, U937 cells, originally derived from a human histiocytic lymphoma and obtained from the American Type Culture Collection (Rockville, MD, USA) and grown in RPMI 1640 medium (Gibco BRL, Grand Island, NY), were labeled for 1 h at 37°C with BCECF/AM (10 mM; Boehringer Mannheim, Mannheim, Germany).Subsequently washed by centrifugation. Confluent A549 cells in 6-well plates were incubated with U937 cells (2×10^6^ cells/ml) at 37°C for 1 h. Non-adherent U937 cells were removed and plates were gently washed twice with PBS. The numbers of adherent U937 cells were determined by counting four fields per 200X high-power field well using a fluorescence microscope (Zeiss, Axiovert 200 M). Experiments were performed in triplicate and repeated at least three times.

### Mouse model and immunohistochemical staining

Male 8-week C57BL/6 mice (n = 12), weighing between 25 and 35 g, were purchased from the National Taiwan University (Taipei, Taiwan) under automatically controlled temperature and light cycle, and were fed standard laboratory chow and tap water, *ad libitum*. All procedures involving experimental animals used in this study were reviewed and approved by the Animal Use Committee of National Taiwan University, indicating that the investigation conforms to the US NIH guidelines for the Care and Use of Laboratory Animals “NIH publication No. 86–23, revised 1985”. Mice were randomly divided into four groups: Saline, TNF-α, TNF-α/CURN, and TNF-α/CURH groups. Before administration of CURN and CURH, CURN powder was added in normal saline and sonicated for 5 min and the preparation of CURH was made by the same method. Mice were anesthetized and intraperitoneally (i.p.) injected with CURN, CURH (200 mg/Kg) or Saline for 1 h. TNF-α (0.75 mg/Kg) was placed posterior in the throat and aspirated into lungs for 24 h. The mice were sacrificed by intraperitoneal injection of an overdose of pentobarbital and the lungs were dissected, immersion-fixed in 4% buffered paraformaldehyde, paraffin-embedded, and then cross-sectioned for immunohistochemstry. The specimens were incubated with antibody against ICAM-1 (1∶100; Santa Cruz, CA) at 4°C for 1 hr, washed with PBS and then incubated with HRP-conjugated goat anti-rabbit IgG (1∶200 dilutions, Sigma). The bound antibody was visualized by using 3, 3′ Diaminobenzidine (Sigma-Aldrich, St. Louis, MO). The other sections were stained with hematoxylin-eosin. In addition, we also determined the curcumin lung tissue distribution after CURN and CURH treatment for verifying curcumin deposited in lung. The lung tissue was homogenized in acetonitrile for extracting curcumin. Curcumin in lung tissue was conducted according to the modified procedures described previously [22]. The HPLC system consisted of a pump (L-2130), a UV-VIS detector (L-2440) and an autosampler (L-2200). The separation was performed by LichroCART Purospher STAR RP-18e (250×4.6 mm i.d., 5 µm) with the mobile phase (10 mM potassium dihydrogen phosphate buffer: acetonitrile, 50∶50). The flow rate was 0.8 mL/min and the UV wavelength was set at 425 nm.

### Analysis of data

All data were estimated and made using a GraphPad Prism Program (GraphPad, San Diego, CA, USA). Data were expressed as the mean±S.E.M. and analyzed by one-way ANOVA followed with Tukey's post-hoc test. *P*<0.05 was considered significant.

## Results

### CURN reduces ICAM-1 expression in TNF-α-treated A549 cells

**Figure 1:**
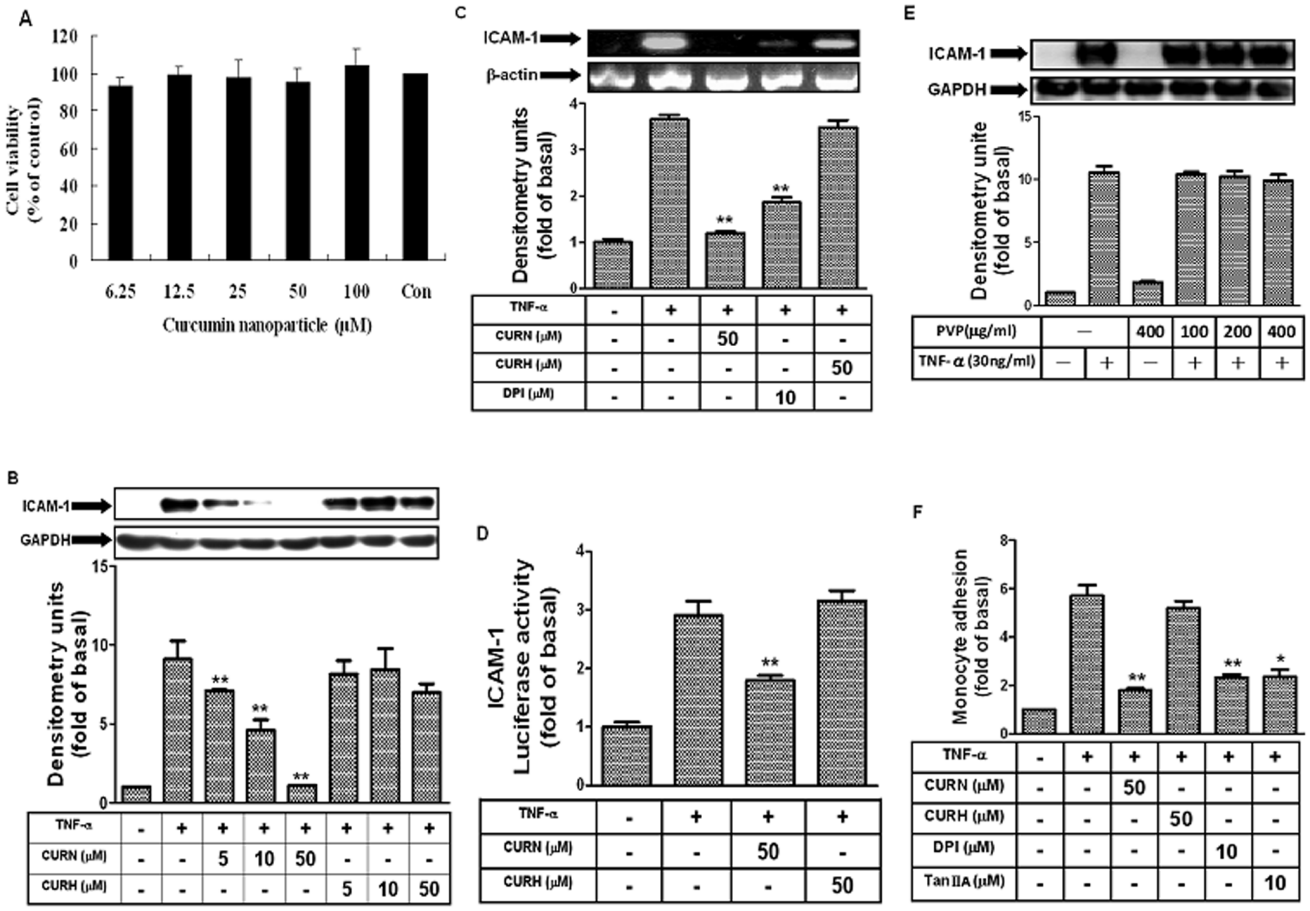
CURN inhibits TNF-α-induced in ICAM-1 gene and protein levels in A549 cells. (**A**) A549 cells were incubated with CURN (0–100 µM) for 24 h, and then the cell viability was determined by MTT assay. (B, E) A549 cells were pretreated with CURN or CURH (5, 10, or 50 µM) or PVP for 1 h, and then incubated with 30 ng/ml TNF-α for 24 h. The levels of ICAM-1 protein were determined by Western blot. (**C**) A549 cells were pretreated with 50 µM CURN, CURH or DPI (10 µM) for 1 h, and then incubated with 30 ng/ml TNF-α for 6 h. The expression of ICAM-1 mRNA was determined by RT-PCR. (**D**) Cells were transiently transfected with ICAM-1-luc reporter gene, pretreated with CURN or CURH for 1 h, and then incubated with TNF-α for 6 h. The ICAM-1 promoter activity was determined in the cell lysates. (**F**) A549 cells were pretreated with 50 µM CURN, CURH, DPI (10 µM) or Tanshinone II A(10 µM) for 1 h, and then incubated with 30 ng/ml TNF-α for 24 h. The U937 cells adherence was measured. The data are expressed as mean±S.E.M. of three independent experiments. ^*^P<0.05; ^**^ *P*<0.01, significant with respect to the basal level.

Cytotoxicity testing of CURN (6.25∼100 µM) in A549 cells using the 3-(4,5-dimethylthiazol-2-yl)-2,5-diphenyltetrazolium bromide assay after 24-h treatment showed no change in cell viability (Fig. 1A). We also compared the effects of CURN and CURH on TNF-α-induced ICAM-1 expression. As shown in Fig. 1B, TNF-α-induced ICAM-1 protein expression was significantly inhibited by the pretreatment with 5, 10, or 50 µM CURN; this response followed a dose-dependent manner. Similar results were also observed in human pulmonary microvascular endothelial cells (data not show). In addition, TNF-α-induced ICAM-1 mRNA expression and promoter activity were also reduced after pretreatment with CURN (Figs. 1C and D). However, these effects were not observed with insoluble curcumin (CURH) and PVP (as a void nanoparticle, Fig. 1E). To further compare the functional consequences of CURN and CURH treatment, we performed a cell adhesion assay using U937 cells as monocytes adhering to A549 cells that were pretreated with TNF-α for 24 h. The number of adherent U937 cells dramatically decreased after pretreatment with CURN, but not with CURH (Fig. 1F). In addition, the adhesion activity was also blocked by DPI (inhibitor of NOX) and tanshinone IIA (inhibitor of AP-1). These results suggested that pretreatment with CURN inhibits TNF-α-induced lung inflammatory responses, which may be regulated by ICAM-1 inhibition. On the other hand, TNF-α-induced ICAM-1 expression might be mediated through NOX and AP-1 in A549 cells and by enhanced monocyte-A549 interactions.

### CURN inhibits TNF-α-induced NADPH oxidase activity and ROS generation

**Figure 2:**
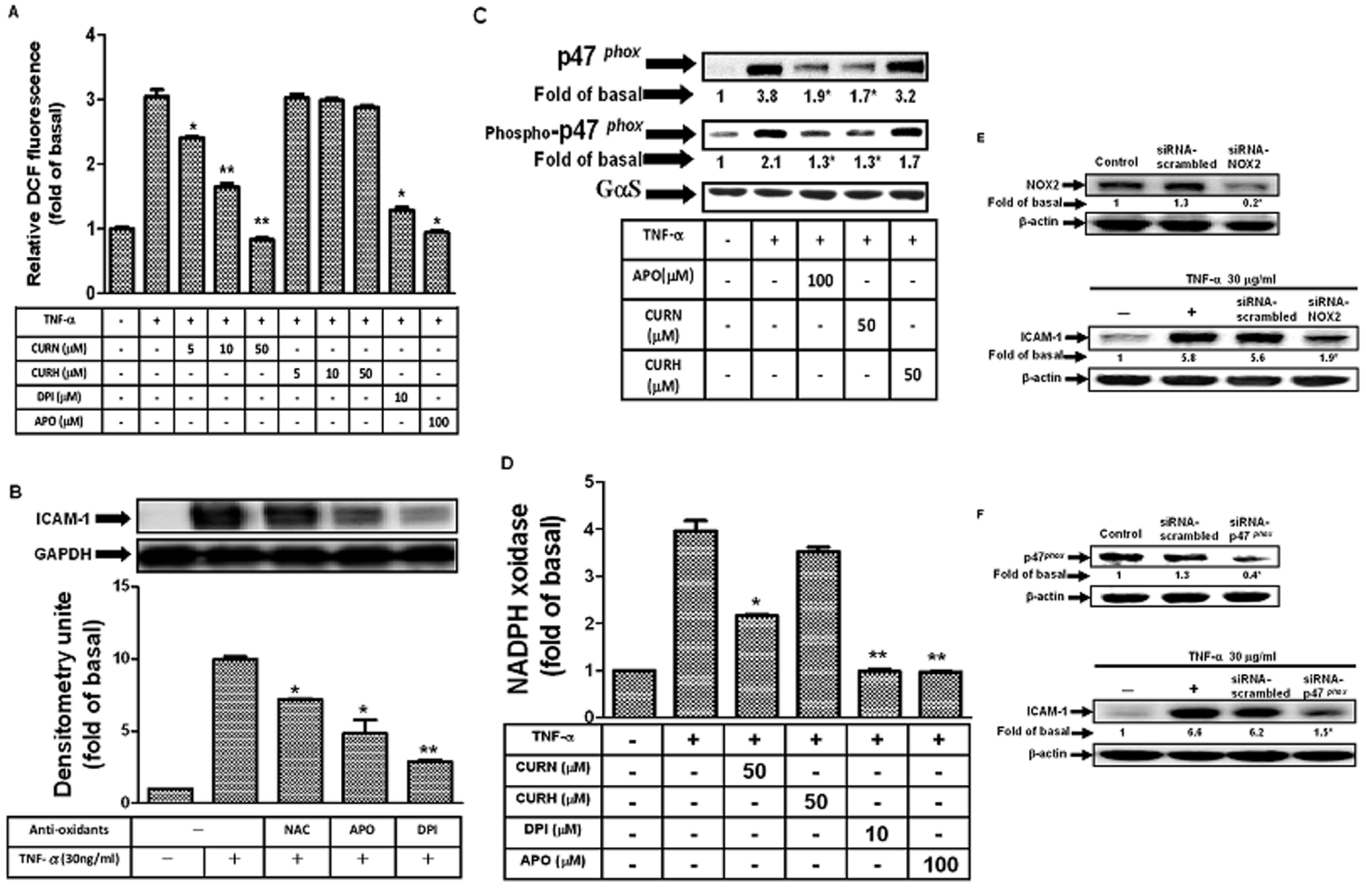
CURN inhibits TNF-α-induced NADPH oxidase activation, ROS generation and p47 *^phox^* translocation in A549 cells. (**A**) Cells were labeled with DCF-DA (10 µM), pretreated with 50 µM CURN, CURH, DPI (10 µM) or APO(100 µM) 1 h, and then incubated with TNF-α for 30 min. The fluorescence intensity (relative DCF fluorescence) was measured. (**B**) A549 cells were pretreated with antioxidants (NAC, APO and DPI) for 1 h, and then incubated with 30 ng/ml TNF-α for 24 h. The levels of ICAM-1 protein were determined by Western blot. (**C**) A549 cells were pretreated with 50 µM CURN, CURH or APO(100 µM) for 1 h, and then incubated with 30 ng/ml TNF-α for 15 min. The membrane fractions were prepared and subjected to Western blot using an anti-p47*^phox^* or anti-phospho-p47*^phox^* antibody. (**D**) A549 cells were pretreated with 50 µM CURN, CURH, 10 µM DPI, and 100 µM APO for 1 h, and then incubated with 30 ng/ml TNF-α for 30 min. The activity of NADPH oxidase was measured. (**E,F**) A549 cells were transfected with scrambled siRNA, p47*^phox^* and NOX2 siRNA, and then incubated with 30 ng/ml TNF-α for 24 h. The levels of ICAM-1 protein were determined by Western blot. The data are expressed as mean±S.E.M. of three independent experiments. ^*^P<0.05; ^**^ *P*<0.01, significant with respect to the basal level.

ROS have been implicated in initiating inflammatory responses in the airway through the activation of transcription factors such as NF-κB and AP-1, leading to an elevated level of expression of adhesion molecules [23], [24]. NOX is an enzymatic source for the production of ROS under various pathologic conditions. To ascertain that the generation of ROS was involved in TNF-α-induced ICAM-1 expression in A549 cells and is affected by CURN, a fluorescent probe DCF-DA was used to determine the generation of ROS in these cells. Cells were labeled with DCF-DA, treated with TNF-α for the 30 min, and the fluorescence intensity (relative DCF fluorescence) was measured at an excitation wavelength of 485 nm and an emission wavelength of 530 nm. As shown in Fig. 2A, TNF-α-induced ROS generation was significantly attenuated by pretreatment with CURN, DPI, and APO (inhibitor of NOX2), whereas no similar effects were observed with CURH. Pretreatment with a ROS scavenger (NAC) or the DPI and APO also markedly inhibited TNF-α-induced ICAM-1 protein expression (Fig. 2B). Moreover, NOX2 consists of the membrane catalytic subunits gp91*^phox^* and p22*^phox^*, together with several cytosolic regulatory subunits, p47*^phox^*, p40*^phox^*, p67*^phox^*, and a small GTPase Rac [9], [11], [25]. The p47*^phox^* regulatory subunit plays a critical role in the acute activation of NOX. In the present study, we showed that TNF-α induced p47*^phox^* phosphorylation and translocation from the cytosol to the membrane within 15 min; such effects were inhibited by CURN and APO (Fig. 2C). However, CURH showed no effects on p47*^phox^* phosphorylation and translocation. Moreover, pretreatment with CURN, DPI, and APO also resulted in a reduction in NADPH oxidase activity in response to TNF-α in A549 cells (Fig. 2D). We finally performed the experiments using siRNAs of NOX2 and p47 *^phox^*. As shown in Figs. 2E and F, transfection with siRNAs of NOX2 and p47 *^phox^* inhibited the expression of ICAM-1 in response to TNF-α. These results thus show that CURN, and not CURH, reduced ICAM-1 expression through the inhibition of NOX2/ROS generation in A549 cells, indicating that CURN exerts better antioxidant activity than CURH.

### CURN reduces TNF-α-stimulated MAPKs phosphorylation

**Figure 3:**
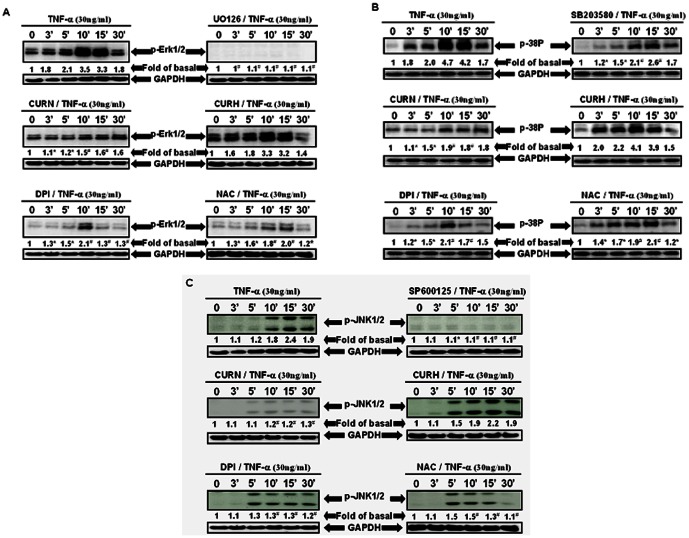
CURN inhibits TNF-α-modulated MAPKs activation in A549 cells. (A–C) Cells were treated with 30 ng/ml TNF-α for the indicated times, then the cell lysate was analyzed for MAPKs phosphorylation by Western blot using antibodies against (A) p-ERK1/2, (B) p-p38, (C) or p-JNK1/2. The cells were preincubated for 1 h with the indicated concentrations of (A) 10 µM U0126 (ERK1/2 inhibitor), (B) 10 µM SB203580 (p38 inhibitor), or (C) 10 µM SP600125 (JNK inhibitor), respectively. (A–C) Western blot analysis also showing the effects of CURN (50 µM), CURH (50 µM) or anti-oxidants treatment on the phosphorylation of p-ERK1/2, p-p38, or p-JNK1/2 in TNF-α-treated A549 cells. Representative results from three separate experiments are shown. ^*^P<0.05; ^#^ *P*<0.01, significant compared with the cells exposed to TNF-α alone group. GAPDH was used as the loading control.

Kinases such as the MAPKs (Erk1/2, p38, and JNK1/2), Akt, Src, and protein phosphatases are components of redox-sensitive signaling pathways and may be targets of NOX-derived ROS [25]–[28]. Thus, the effects of CURN pretreatment on TNF-α-induced MAPK phosphorylation were compared with that of CURH. As shown in Figs. 3A–C, the phosphorylation of Erk1/2, p38, and JNK1/2 was significantly attenuated by U0126, SB202190, and SP600125, respectively. Furthermore, pretreatment with CURN, NAC and DPI also resulted in a significant decrease in TNF-α-induced MAPKs phosphorylation, whereas no similar effects were observed with that using CURH. These results suggest that CURN imparts stronger inhibitory effects than CURH on TNF-α-induced MAPKs phosphorylation, which resulted from the inhibition of the NOX2/ROS-dependent pathway in A549 cells.

### CURN decreases AP-1 activation in TNF-α-treated A549 cells

**Figure 4:**
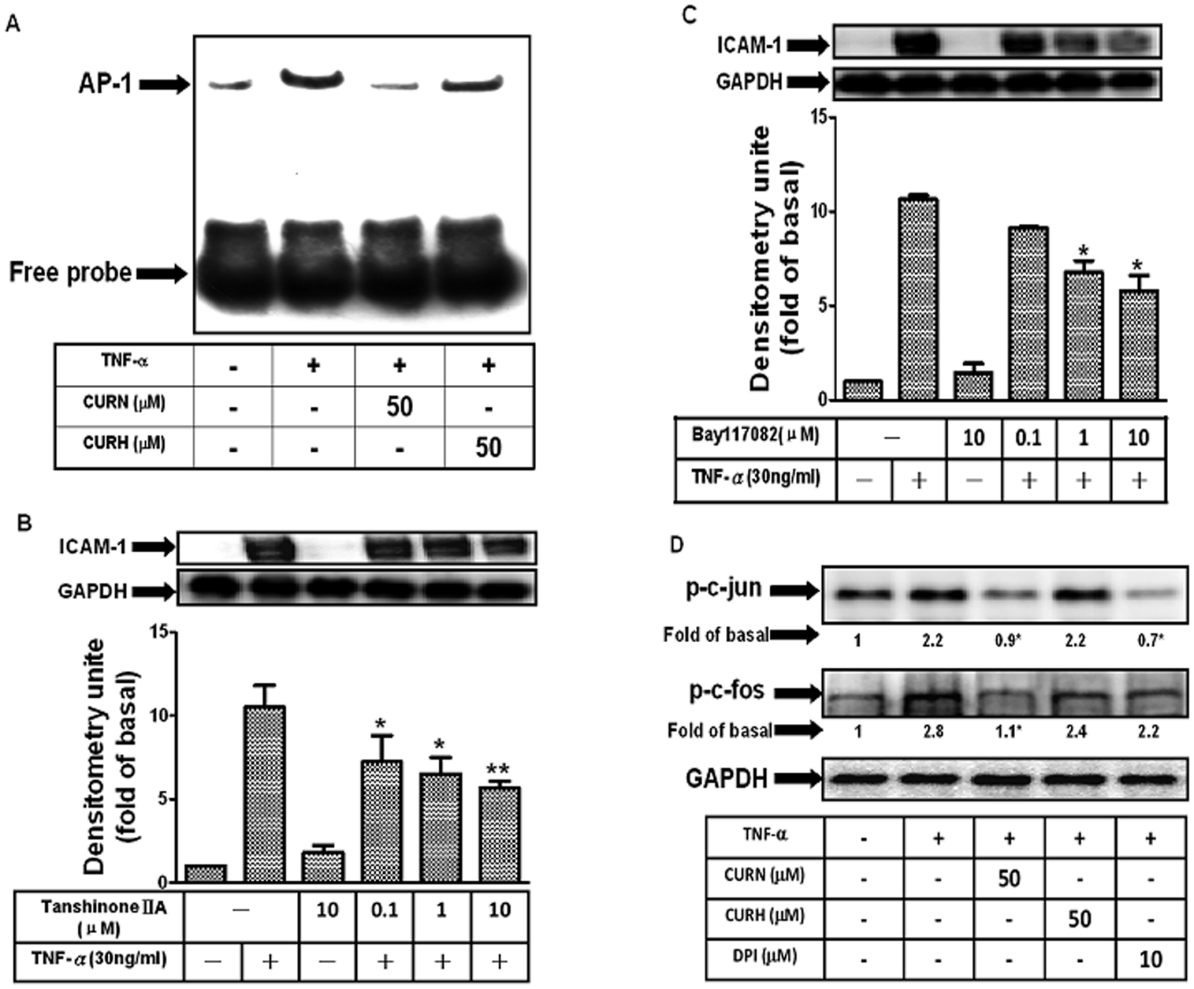
The CURN caused down-regulation of ICAM-1 expression in TNF-α-stimulated A549 is mediated by inhibition of AP-1 activation. (A) Nuclear extracts prepared from untreated cells or from cells with or without 24 h pretreatment with 50 µM CURN or CURH and then incubated with 30 ng/ml TNF-α for 1 h were tested for (A) AP-1 and NF-κB DNA binding activity by EMSA. (B, C) Cells were pretreated with Tanshinone II A(AP-1 inhibitor) and Bay117082 (NF-κB inhibitor) for 1 h, and then incubated with 30 ng/ml TNF-α for 24 h. The levels of ICAM-1 protein were determined by Western blot. (D) Cells were pretreated with 50 µM CURN, CURH or 10 µM DPI for 1 h, and then incubated with 30 ng/ml TNF-α for 30 min. The levels of phosphorylation of c-jun and c-fos were determined by Western blot. Representative results from three separate experiments are shown. The data are expressed as a fold of the control value and are the means±SEM for three separate experiments. GAPDH was used as the loading control.

Because the ICAM-1 gene promoter contains consensus binding sites for AP-1 and NF-κB [29], [30], we investigated whether CURN inhibited TNF-α-induced ICAM-1 expression through its transcription factors, and compared these effects with that using CURH. Gel-shift assays were performed to determine the effects of CURN and CURH on AP-1 and NF-κB activation in TNF-α-treated A549 cells. As shown in Fig. 4A, low basal levels of AP-1 binding activity were detected in untreated control cells, whereas binding was significantly increased after TNF-α treatment for 30 min. Pretreatment with CURN for 24 h inhibited an increase in AP-1 binding activity, whereas no similar results were observed in that using CURH. TNF-α also enhanced the NF-κB binding activity, although no differences were observed using CURN and CURH (data not shown). Pretreatment with inhibitors of NF-κB (Bay117082) or AP-1 (Tanshinone IIA) resulted in a significant decrease in TNF-α-induced ICAM-1 expression; this response occurred in a dose-dependent manner (Figs. 4B and C). In addition, the AP-1 transcriptional complex is composed of at least two major factors, c-Fos and c-Jun. The phosphorylation of c-Fos and c-Jun were detected under TNF-α stimulation (Fig. 4D). Pretreatment with CURN and DPI resulted in a significant decrease in c-Fos and c-Jun phosphorylation, whereas no similar effects were observed with CURH. These results suggest that CURN imparts a greater inhibitory effect on the TNF-α-induced ICAM-1 expression by blocking AP-1 activation.

### CURN reduces ICAM-1 protein expression in the lung alveolar epithelial cells in TNF-α-injected mice

**Figure 5:**
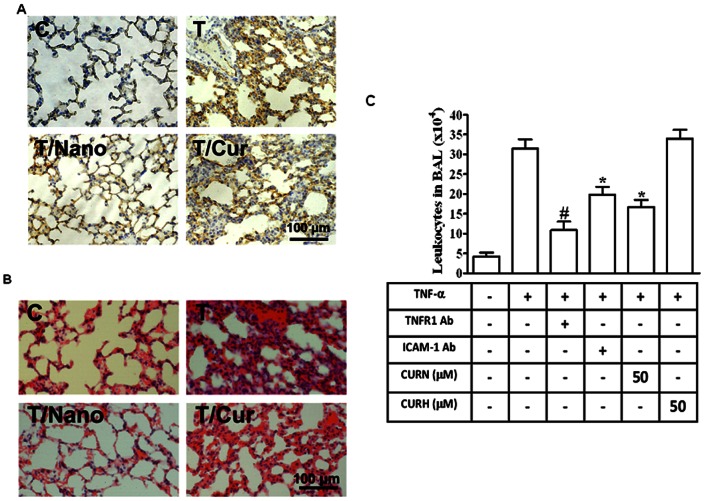
CURN prevents TNF-α-induced acute lung inflammation *in vivo*. Mice were intraperitoneally injected with CURN or CURH (200 mg/Kg) for 1 h followed by acute challenged with TNF-α(0.75 mg/Kg) by laryngoscopy for 24 h. The sections were stained with ICAM-1 (A) and hematoxylin-eosin (B). Higher magnification images were obtained under microscope at 400X magnification. Bar = 100 µm. (C) Mice were intraperitoneally given one dose of TNFR1 antibody, ICAM-1 antibody, CURN and CURH for 1 h prior to TNF-α (0.75 mg/kg) treatment and sacrificed after 24 h. BAL fluid was acquired, and leukocyte count was determined by a hemocytometer. Representative results from three separate experiments are shown.

To determine the anti-inflammatory effects of curcumin nanoparticles *in vivo*, mice were intraperitoneally injected with CURN or CURH (200 mg/kg) for 1 h, and then treated with TNF-α(0.75 mg/Kg) for 24 h. As illustrated in Fig. 5A, TNF-αsignificantly induced ICAM-1 expression (brown color), whereas injection with CURN, but not CURH, effectively decreased TNF-α-induced ICAM-1 expression in the alveolar walls. Histological examination showed a TNF-αinduced thickening of the alveolar walls, increased amount of alveolar hemorrhage, and a greater number of infiltrating inflammatory cells (Fig. 5B). Moreover, we have also performed a bronchoalveolar lavage (BAL) to measure PMN influx. As shown in Fig. 5C, TNF-α significantly enhanced ICAM-1 expression and leukocyte (eosinophils and neutrophils) count in the BAL fluid in mice, which were attenuated by intraperitoneally of TNFR1-antibody, ICAM-1-antibody, CURN, but not by CURH. In contrast, preadministration of CURN showed a greater inhibitory effect on lung inflammation by decreasing ICAM-1 staining in the TNF-α-treated animals.

### CURN distribution in the lung is higher, suggesting a better anti-inflammatory effect

**Figure 6:**
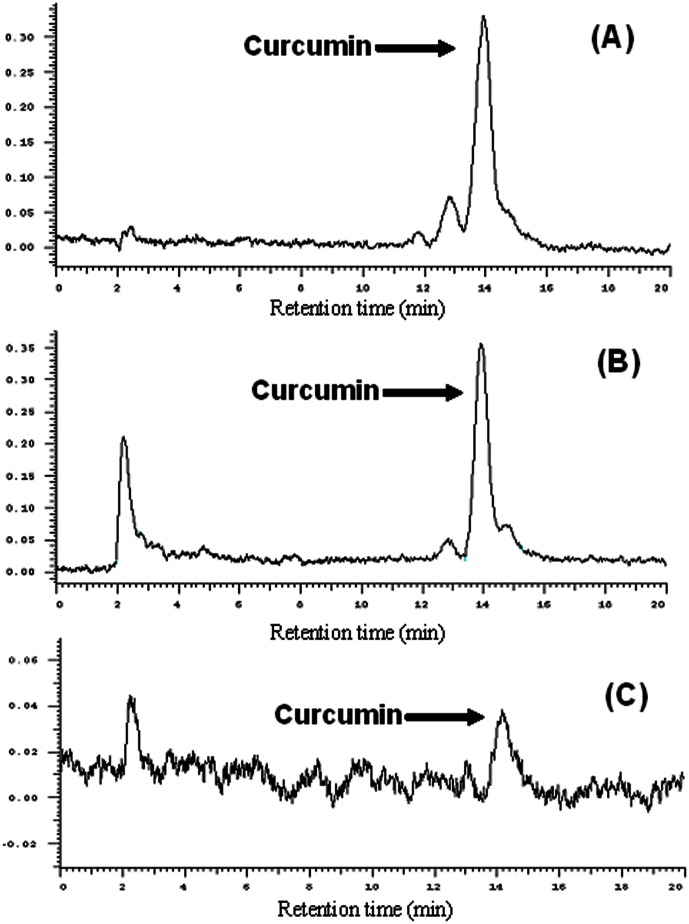
Lung distribution of CURN and CURH. HPLC chromatographs of (A) blank lung with curcumin, (B) CURN in lung, (C) CURH in lung.

Our results indicate that CURN has better anti-inflammatory effects compared to that observed with CURH. To confirm curcumin distribution in the lung tissue of a single dose of CURN and CRUH in mice with TNF-α-induced lung injury, we used the HPLC analysis to determine the curcumin level of CURN and CURH in the lung tissues. As shown in Fig. 6, curcumin distribution in the lung tissue and the curcumin level is 934.9±72.1 ng/gm. The HPLC chromatography of CURH-treated lung tissue only showed a curcumin signal, whereas the curcumin level was below the detection limit. Based on these findings, CURN can distribute greater amounts of curcumin in the lung tissues as compared to CURH. It has also been determined that the curcumin levels in the lung tissues were higher with CURN, as compared to that using CURH (Table 1). These findings indicate that CURN can increase curcumin uptake in lung cells, and result in better antioxidant and anti-inflammatory activities compared to that using CURH.

**Table 1:** The curcumin level in lung tissue after CURN and CURH treatment in mice.

Group	Curcumin level in lung (ng/gm)
CURH	BDL*
CURN	934.9±72.1

BDL* below the detection limit in lung tissue (20 ng/gm)

## Discussion

Dietary polyphenols such as curcumin could be effectively used to prevent aggravation in many diseases by virtue of their biological and pharmacological activities. It is commonly used as a spice, food preservative, and nontoxic agent. Extensive research lasting the past five decades has revealed that curcumin possess anti-oxidant, anti-inflammatory, anti-cancerous, anti-microbial, anti-parasitic, anti-cholesterol, anti-mutagenic properties, wound-healing properties, cardiovascular, and hepatic and neuronal protective activities [18], [31]–[33]. However, the low level of water solubility of curcumin has become a major limiting factor in preclinical dissolution testing for drug and health food applications. Thus, for curcumin to exhibit its therapeutic effects in the human body, a person is required to swallow between 12 and 20 g of curcumin per day. For decades, nanotechnology platforms have been widely used to create delivery systems for natural products and nutraceuticals with poor water solubility [15], [17], [34]. Our previous study utilized PVP in the development of CURN. Previous reports have described the enhanced antioxidant and anticancer effects of CURN in hepatocytes compared to curcumin water preparations [19]. In the current study, we analyzed the inhibitory effects of CURN on ICAM-1 expression both *in vitro*, in TNF-α-stimulated lung epithelial cells, and *in vivo*, in the lung epithelium of TNF-α-treated mice; these effects were also compared to that using CURH and in relation to the ROS-derived MAPKs pathways. The results of our study showed that CURN decreased the expression of ICAM-1, inhibited NADPH oxidase (NOX)-derived ROS generation, and reduced MAPKs and AP-1 transcription factor binding activities.

Comparative analysis of the anti-inflammatory effects of CURN and CURH on lung epithelial cells showed a significantly greater inhibition of ICAM-1 expression in both gene and protein levels (Figs. 1B–D). However, TNF-α-induced ICAM-1 expression was not attenuated by CURH and PVP (as a void nanoparticle, Fig. 1E). In the functional activity assay, CURN showed an inhibition in the binding activity of the human histiocytic lymphoma cell line U937 to TNF-α-stimulated lung epithelial cells; a similar effect was not observed using CURH treatment (Fig. 1F). *In vivo* study performed in BAL fluid isolation in mice also showed that CURN reduced PMN influx, such effect was also shown on the treatment with antibodies of TNFR1 and ICAM-1 (Fig. 5C). Although the CURN treatment results in a decrease in the ICAM-1 expression (Fig. 1B), it only partially affects monocyte adhesion (Fig. 1F) and leukocyte infiltration (Fig. 5C). Thus, other factors may also be associated with ICAM-1 expression, such as vascular adhesion molecule (VCAM-1) [35] or selectin [36]. The putative functional groups of curcumin, a benzene ring with adjacent methoxy-hydroxyl groups, serve as a potent inhibitor of NOX activity [37]. Interestingly, pretreatment with DPI (inhibitor of NOX) caused a significant reduction in TNF-α-enhanced monocyte-A549 interactions. These results suggest that CURN inhibits TNF-α-induced ICAM-1 expression through more potent antioxidative properties. Further, we compared with the antioxidant activity of CURN and CURH on the expression of ICAM-1 upon TNF-α stimulation. When lung tissues are exposed to oxidative stress, NOX isoforms represent a major source of ROS within the respiratory system. ROS have also been shown to mediate the expression of VCAM-1 and ICAM-1 in airways [38]. In our present data, pretreatment with CURN, DPI, and APO markedly reduced both ROS generation and NOX activation; similar effects were not observed with CURH treatment (Figs. 2A and D). In addition, antioxidants (NAC, DPI, and APO) also inhibited TNF-α-induced ICAM-1 expression, suggesting that ROS regulated the effects of TNF-α-induced ICAM-1 expression (Fig. 2D). NOX is a multi-subunit enzymatic complex responsible for the production of superoxide caused by the monoelectronic reduction of oxygen using NADPH or NADH. In addition, NOX2 is robustly expressed in lung alveolar epithelial cells and involved in the regulation of epithelial cell function [39]. Here, we generated robust evidence that transfection with siRNAs of NOX2 and p47 *^phox^* can block the expression of ICAM-1 in response to TNF-α (Figs. 2E and F). Although the NOX2 plays a critical role in lung inflammation, which may be influenced by ICAM-1 expression, the epithelial cells also expressed other species of NOX, such as NOX1 and NOX4. Both NOX1 and NOX4 are key players in epithelial cell death, leading to pulmonary fibrosis [40] and acute lung injury [41]. Whether NOX1 and NOX4 are involved in the TNF-α-induced lung inflammation needs to be investigated in the future. Moreover, phosphorylation of p47*^phox^* acts as the organizer of NOX2 complex by facilitating the assembly, translocation, and binding of the cytosolic subunits to the gp91*^phox^*/p22*^phox^* catalytic complex. We therefore compared with the effect of CURN and CURH on the activation of p47*^phox^*. As shown in Fig. 2C, CURN and APO have more potent inhibitory effects on the p47*^phox^* activation, which included phosphorylation and translocation from the cytosol to the membrane within 15 min of TNF-α treatment. In contrast, CURH showed no effects on p47*^phox^* phosphorylation and translocation. These results suggest that CURN reduced ICAM-1 expression through the inhibition of NADPH oxidase activity and p47 *^phox^* activation in the membrane fraction of TNF-α-treated A549 cells, thus exerting stronger antioxidant activity than CURH.

In previous studies, ROS has been shown to modulate downstream signal transduction, such as MAPKs. The MAPKs, which consist of three major members, namely, ERK, p38, and JNK, are a family of serine/threonine kinases that control cellular responses to growth, apoptosis, inflammation, and stress signals [42], [43]. The upregulation of ICAM-1 by cytokines has been shown to be mediated through activation of MAPKs in A549 cells [44]. This study compared with the effects of CURN and CURH on the phosphorylation of MAPKs. We have found that pretreatment with U0126, SB202190, and SP600125 resulted in a decrease in TNF-α-induced phosphorylation of Erk1/2, p38, and JNK1/2, respectively (Figs. 3A–C). Additionally, we also demonstrated that ROS was involved in TNF-α-induced MAPKs phosphorylation after cells were treated with antioxidants, NAC, and DPI. Our data also showed that CURN, but not CURH, markedly inhibited TNF-α-induced MAPKs phosphorylation, which may be mediated through its potent antioxidant activities.

MAPKs have been shown to phosphorylate transcription factors or intracellular enzymes, including NF-κB, AP-1, ATF2, and Elk-1 [45]. Transcription factors such as NF-κB and AP-1 have been shown to regulate VCAM-1 and ICAM-1 expression induced by TNF-α [8], [20]. Thus, we first confirmed the involvement of NF-κB and AP-1 in TNF-α-induced ICAM-1 expression by incubation with inhibitors of Bay117082 and Tanshinone IIA as shown in Figs. 4B and C. Moreover, gel-shift assays demonstrated that binding of NF-κB and AP-1 to the ICAM-1 promoter was increased by TNF-α and that pretreatment with CURN significantly attenuated TNF-α-induced AP-1 binding activity (Fig. 4A), but not NF-κB (data not shown). The possible mechanism underlying the effect of curcumin on NF-κB inhibition remains to be investigated. Furthermore, AP-1 functions as a dimer in the form of c-Fos/c-Jun or c-Jun/c-Jun. We examined the effect of CURN or CURH on c-Fos and c-Jun phosphorylation. As shown in Fig. 4D, both c-Fos and c-Jun phosphorylation were markedly inhibited by CURN and DPI; such effects were not found after treatment with CURH. Based on our findings and previous reports, CURN shows greater inhibitory effects than CURH on TNF-α-induced ICAM-1 expression and this is attributable to the inhibition of the NOX2/ROS/MAPKs/AP-1-dependent pathway in A549 cells.

In addition to the *in vitro* studies, our previous results have shown that the PVP-loaded novel nanoparticle system enhanced the dissolution properties of curcumin by reducing its particle size and by improving its physicochemical characteristics, which are crucial factors for achieving optimal *in vivo* efficacy [19]. Therefore, the present study also measured the curcumin levels in the lungs to confirm its distribution after CURN and CURH treatment. Our results (Table 1) demonstrated that intraperitoneally injected CURN results in a higher curcumin level distribution in the lungs as compared to that using CURH. These findings suggest that CURN can increase the cell uptake in lungs and then show better antioxidant and anti-inflammatory activities than CURH. In the present study, examination of an animal model of TNF-α-induced ICAM-1 expression and inflammatory cells infiltration established the superiority of CURN over CURH in the pulmonary protective effect (Figs. 5A and B); this may be attributable to its effective antioxidant and anti-inflammatory activities. Consequently, we suggest that CURN deserves further study and may potentially be used during prophylaxis of inflammatory pulmonary diseases.
